# Protecting the heart in cancer therapy

**DOI:** 10.12688/f1000research.15190.1

**Published:** 2018-09-28

**Authors:** J. Emanuel Finet, W. H. Wilson Tang

**Affiliations:** 1 Section of Heart Failure and Transplantation Medicine, Robert and Suzanne Tomsich Department of Cardiovascular Medicine, and Sydell and Arnold Miller Family Heart and Vascular Institute, Cleveland Clinic, Cleveland, USA; 2 Cleveland Clinic Lerner College of Medicine at Case Western Reserve University; Center for Clinical Genomics; Cleveland Clinic, Cleveland, USA

**Keywords:** Cardiovascular disease, cancer, heart, cardioprotection, cardiotoxicity, prevention, biomarkers

## Abstract

Recent advances in cancer prevention and management have led to an exponential increase of cancer survivors worldwide. Regrettably, cardiovascular disease has risen in the aftermath as one of the most devastating consequences of cancer therapies. In this work, we define cancer therapeutics-induced cardiotoxicity as the direct or indirect cardiovascular injury or injurious effect caused by cancer therapies. We describe four progressive stages of this condition and four corresponding levels of prevention, each having a specific goal, focus, and means of action. We subsequently unfold this didactic framework, surveying mechanisms of cardiotoxicity, risk factors, cardioprotectants, biomarkers, and diagnostic imaging modalities. Finally, we outline the most current evidence-based recommendations in this area according to multidisciplinary expert consensus guidelines.

## Introduction

Recent advances in cancer prevention and management have led to an exponential increase of cancer survivors worldwide ^1^. Regrettably, cardiovascular disease (CVD) has risen in the aftermath as one of the most devastating consequences of cancer therapies ^2, 3^, being most prevalent in adult survivors of breast cancer and hematological malignancies ^1, 4, 5^.

In this work, we define cancer therapeutics-induced cardiotoxicity (CTIC) as the direct or indirect cardiovascular injury or injurious effect caused by cancer therapies, such as mediastinal radiotherapy ^6^ and/or some chemotherapeutic agents ^7^. These incipient toxic changes (e.g. cardiomyocyte apoptosis, cardiac ion-channel alteration, endothelial damage, etc.) can further develop into complex cardiovascular conditions, such as heart failure (HF), valvular heart disease, coronary artery disease (CAD), pericardial disease, systemic and pulmonary hypertension, arrhythmias, and thromboembolic disease, among others ^8, 9^. Concomitant pre-existent cardiovascular risk factors have been shown to foment this pathogenesis ^10^.

## Pathogenesis of cancer therapeutics-induced cardiotoxicity

### Cardiotoxic chemotherapy

Doxorubicin (and other agents in the anthracycline family) is the archetype chemotherapeutic leading to CTIC, historically called anthracycline-induced cardiotoxicity or anthracycline-induced cardiomyopathy (AIC) ^11^. The hallmark of this condition is a HF syndrome arising from dilated cardiomyopathy (DCM) ^11^; supraventricular and ventricular arrhythmias have also been described during anthracycline administration but seldom require intervention ^12^. Its prevalence has not been thoroughly studied owing to lack of a uniform definition, inconsistent diagnostic criteria, and underreporting; in modern times, it is thought to affect 17–23% of survivors of pediatric hematological malignancies ^13– 15^ and accounts for 2.6% of all patients with non-ischemic cardiomyopathy undergoing cardiac transplantation ^16^.

In addition to anthracyclines, an increasing number of chemotherapeutic agents have been labeled as “cardiotoxic”, with particular mechanisms of action that lead to distinctive cardiovascular effects, and in turn various degrees of frequency and severity (see Table 1 for a list of the most important cardiotoxic chemotherapeutic agents currently available in the US) ^7, 8, 17^. Because historical cardiotoxicity was mediated by non-specific agents such as anthracycline and alkylating agents, it was believed that the novel “targeted therapeutics” (e.g. monoclonal antibodies, tyrosine kinase inhibitors, etc.) would provide fewer off-target adverse effects. However, an increasingly systematic evaluation and reporting of cardiovascular safety, along with a concomitant explosion of basic ^18^, translational ^19^, and clinical research in the area of CTIC ^20^, have progressively revealed that a large number of these targeted agents are mechanistically determined to cause cardiotoxicity ^21^. Based on the weight of the evidence, the US Food and Drug Administration has recently issued several cardiovascular box warnings for some of these agents, such as myocardial toxicity for anthracyclines, cardiomyopathy for ERBB2 inhibitors, QT prolongation and sudden cardiac death for certain tyrosine kinase inhibitors, and immune-mediated adverse reactions (i.e. myocarditis) for CTLA-4 inhibitors, among others (see Table 1) ^17^.

**Table 1.** Chemotherapy agents associated with cancer therapeutics-induced cardiotoxicity. Text in bold represents US Food and Drug Administration box warnings. 5-FU, 5-fluorouracil; ALK, anaplastic lymphoma kinase; CSF-1R, colony-stimulating factor 1 receptor; ECG, electrocardiogram; EGFR, epidermal growth factor receptor; FKBP, FK506-binding protein; FGFR, fibroblast growth factor receptor; FLT3, FMS-like tyrosine kinase 3; GIST, gastrointestinal stromal tumor; GVHD, graft-versus-host disease; LT3, Lymphotoxin 3; HDAC, histone deacetylase; HGFR, hepatocyte growth factor receptor; HIF-1, hypoxia-inducible factor-1; Ig, immunoglobulin; IGF-1R, insulin-like growth factor 1-receptor; IL, interleukin; LAK, lymphokine-activated killer; mTOR, mammalian target of rapamycin; NK, natural killer; PD-1, programmed death 1; PDGFR, platelet-derived growth factor receptor; PD-L1, programmed death ligand 1; PNET, primitive neuroectodermal tumor; SCD, sudden cardiac death; TdP, Torsades de Pointes; TIL, tumor-infiltrating lymphocyte; VEGF; vascular endothelial growth factor; VEGFR, vascular endothelial growth factor receptor.

Chemotherapy agents associated with cancer therapeutics-induced cardiotoxicity				
Family	Agent	Approved uses	Mechanism of action	Cardiovascular toxicities
Anthracyclines	Doxorubicin	*Breast cancer, non-Hodgkin lymphoma, Burkitt* *lymphoma, mantle cell lymphoma, Hodgkin* *lymphoma, Waldenstrom macroglobulinemia,* *acute lymphocytic leukemia, small cell lung cancer, multiple* *myeloma, gastric cancer, bladder cancer, Wilms’* *tumor, bone sarcoma, soft tissue sarcoma, thymoma,* *neuroblastoma, hepatoblastoma, endometrial cancer*	Anthracyclines bind directly to DNA (intercalation) and also inhibit DNA repair (via topoisomerase II inhibition), resulting in blockade of DNA and RNA synthesis and fragmentation of DNA. Doxorubicin is also a p53 inhibitor and powerful iron chelator; the iron–doxorubicin complex binds to DNA and cell membranes, producing free radicals that cleave the DNA and cell membranes.	Acute myocarditis, cardiomyopathy, heart failure, bradyarrhythmias and tachyarrhythmias, non-specific ST or T wave changes. **BOX WARNING:** **MYOCARDIAL TOXICITY**
	Daunorubicin	*Acute myelocytic leukemia, acute lymphocytic* *leukemia, Kaposi sarcoma, non-Hodgkin lymphoma*		
	Idarubicin	*Acute promyelocytic leukemia, acute myelocytic* *leukemia*		
	Epirubicin	*Breast cancer, soft tissue sarcoma, bone sarcoma,* *gastric cancer, esophageal cancer*		
	Mitoxantrone	*Non-Hodgkin lymphoma, Hodgkin lymphoma,* *prostate cancer, breast cancer, acute promyelocytic* *leukemia, acute myelocytic leukemia*		
Alkylating agents	Cyclophosphamide	*Breast cancer, non-Hodgkin lymphoma, mantle cell* *lymphoma, follicular lymphoma, Burkitt lymphoma,* *Hodgkin lymphoma, Waldenstrom macroglobulinemia,* *acute lymphocytic leukemia, small cell lung cancer,* *lymphoma, AL amyloidosis, multiple myeloma, gastric* *cancer, esophageal cancer, soft tissue sarcoma,* *Wilms’ tumor, gestational trophoblastic tumor,* *neuroblastoma, bone sarcoma, brain tumor, ovarian* *cancer, thymoma*	Alkylating agents prevent cell division by cross-linking DNA strands and binding with nucleic acids and other intracellular structures, inhibiting protein synthesis and DNA synthesis, resulting in cell death.	Atrial tachyarrhythmias or bradyarrhythmias, capillary leak syndrome, cardiac arrest, cardiomyopathy, heart failure, cardiogenic shock, hemopericardium, hemorrhagic myocarditis.
	Ifosfamide	*Hodgkin lymphoma, non-Hodgkin lymphoma, Burkitt* *lymphoma, neuroblastoma, small cell lung cancer,* *penile cancer, testicular cancer, hepatoblastoma,* *bone sarcoma, soft tissue sarcoma*		
	Mitomycin	*Gastric cancer, anal cancer, pancreatic cancer, lung* *cancer, mesothelioma, bladder cancer, breast cancer*		
	Bleomycin	*Hodgkin lymphoma, testicular cancer, ovarian cancer*		Phlebitis, pericarditis, chest pain, myocardial ischemia
	Cisplatin	*Bladder cancer, ovarian cancer, testicular cancer,* *breast cancer, cervical cancer, endometrial cancer,* *esophageal cancer, gastric cancer, head and neck* *cancer, Hodgkin lymphoma, mesothelioma, non-* *Hodgkin lymphoma, non-small cell lung cancer,* *osteosarcoma, penile cancer, small cell lung cancer*		Arrhythmias, myocardial ischemia and infarction, ischemic cardiomyopathy, Raynaud’s phenomenon, hypertension, stroke
	Trabectedin	*Soft tissue sarcoma, ovarian cancer*		Cardiomyopathy, heart failure, cardiac arrest, peripheral edema, pulmonary embolism
Antimetabolites	5-FU	*Breast cancer, anal cancer, gastric cancer,* *esophageal cancer, colorectal cancer, cervical* *cancer, bladder cancer, head and neck cancer,* *pancreatic cancer*	Antimetabolites inhibit DNA polymerase, interfering with DNA and, to a lesser degree, RNA synthesis. Some agents also inhibit ribonucleotide reductase, DNA primase, and DNA ligase I.	Angina pectoris, vasospasm, myocardial infarction, non-specific ECG changes, atrial and ventricular bradyarrhythmias and tachyarrhythmias, cardiomyopathy, heart failure, pericardial effusion, cerebrovascular accident, local thrombophlebitis, pericarditis
	Capecitabine	*Colorectal cancer, breast cancer, biliary cancer,* *esophageal cancer, pancreatic cancer, gastric cancer*		
	Fludarabine	*Chronic lymphocytic leukemia, acute myeloid* *leukemia, hematopoietic stem cell transplant, non-* *Hodgkin lymphoma, Waldenstrom macroglobulinemia*		
	Cytarabine	*Acute myelocytic leukemia, acute promyelocytic* *leukemia, acute lymphocytic leukemia, chronic* *lymphocytic leukemia, primary central nervous* *system lymphoma, Hodgkin lymphoma, non-Hodgkin* *lymphoma, meningeal leukemia*		
Anti-ERBB monoclonal antibodies	Trastuzumab	*Breast cancer and gastric cancer (ERBB2+)*	Binds to ERBB1 (EGFR) or ERBB2 (HER- 2), mediating antibody-dependent cellular cytotoxicity of cells that overexpress EGFR or HER-2 proteins.	Cardiomyopathy, heart failure, peripheral edema, hypertension, arrhythmias. **BOX** **WARNING: CARDIOMYOPATHY**
	Pertuzumab			
	Necitumumab	*Non-small cell lung cancer (ERBB1+)*		Arrhythmias, venous and arterial thomboembolism, ischemia. **BOX** **WARNING: CARDIOPULMONARY** **ARREST**
Anti-VEGF monoclonal antibodies	Bevacizumab	*Non-small cell lung cancer, cervical cancer, ovarian* *cancer, breast cancer, endometrial cancer, renal cell* *cancer, glioblastoma, soft tissue sarcoma, colorectal* *cancer*	Binds to and neutralizes VEGF-A, preventing its association with the endothelial receptors VEGFR1 and VEGFR2, inhibiting angiogenesis and thus retarding the growth of all tissues (including metastatic tissue).	Hypertension, cardiomyopathy, heart failure, peripheral edema, hypotension, venous and arterial thromboembolism, syncope, pulmonary embolism
	Aflibercept	*Colorectal cancer*	Inhibits VEGFR1 and VEGFR2	
	Ramucirumab	*Colorectal cancer, gastric cancer, non-small cell lung* *cancer*	Inhibits VEGFR2	
Immune checkpoint inhibitors (monoclonal antibodies)	Ipilimumab	*Melanoma, small cell lung cancer*	Human IgG1 that blocks CTLA-4, which is a downregulator of T-cell activation pathways, enhancing their activation and proliferation	Acute myocarditis, cardiogenic shock. **BOX WARNING: IMMUNE-MEDIATED** **ADVERSE REACTIONS (including** **autoimmune myocarditis)**
	Nivolumab	*Head and neck cancer, Hodgkin lymphoma,* *melanoma, non-small cell lung cancer, renal cell* *cancer, urothelial carcinoma, small cell lung cancer*	Human IgG4 that inhibits PD-1, enhancing T-cell activation and proliferation. It potentiates the effects of CTLA-4 inhibitors	Peripheral edema, acute myocarditis, cardiogenic shock, pulmonary embolism
	Pembrolizumab			
	Atezolizumab	*Non-small cell lung cancer, urothelial carcinoma*	Human IgG1 that inhibits PD-L1 and CD80, enhancing T-cell activation and proliferation. It potentiates the effects of CTLA-4 inhibitors	Peripheral edema, venous thromboembolism
	Avelumab	*Merkel cell carcinoma, urothelial carcinoma*		Peripheral edema, hypertension
	Durvalumab	*Urothelial carcinoma*		Peripheral edema, myocarditis
Multi-targeted (VEGFR) tyrosine kinase inhibitors	Sunitinib	*Renal cell cancer, soft tissue sarcoma, GIST*	Inhibits multiple receptor tyrosine kinases (VEGFR1, VEGFR2, and VEGFR3 mainly; also inhibits PDGFRα/β; LT3; FLT3; CSF- 1R; RET; FGFR-1/3; cKIT; IL-2R; Lck; c- Fms; RET/PTC; CRAF; BRAF), preventing tumor growth and angiogenesis.	Hypertension, QTc prolongation, bradycardia, peripheral edema, cardiomyopathy, heart failure, chest pain, venous and arterial thromboembolim, ischemia, myocardial infarction, arrhythmias. **BOX WARNING: QTc** **PROLONGATION, TdP, AND SCD** **(vandetanib)**
	Pazopanib	*Renal cell cancer, soft tissue sarcoma, thyroid cancer*		
	Sorafenib	*Renal cell cancer, hepatocellular cancer, soft tissue sarcoma, GIST, thyroid cancer*		
	Axitinib	*Renal cell cancer, thyroid cancer*		
	Lenvatinib	*Renal cell cancer, thyroid cancer*		
	Regorafenib	*Colorectal cancer, GIST, hepatocellular carcinoma*		
	Vandetanib	*Thyroid cancer (medullary)*		
Multi-targeted (BCR-ABL) tyrosine kinase inhibitors	Imatinib	*Acute lymphocytic leukemia, acute myelocytic* *leukemia, GIST*	Inhibits multiple receptor tyrosine kinases (Bcr-Abl mainly; also VEGFRs, PDGFRβ; SRC; LCK; YES; FYN; cKIT; EPHA2, among others), inducing apoptosis.	Edema (anasarca, ascites, pericardial and pleural effusion, peripheral edema, pulmonary edema, and superficial edema), hypertension, hypotension, chest pain, cardiomyopathy, heart failure, QTc prolongation, tachyarrhythmias and bradyarrhythmias, pulmonary hypertension, myocardial ischemia and infarction. **BOX WARNING: QTc** **PROLONGATION, TdP, AND SCD** **(nilotinib). BOX WARNING: HEART FAILURE; ARTERIAL AND VENOUS** **THROMBOEMBOLISM (ponatinib)**
	Dasatinib	*Acute lymphocytic leukemia, chronic myelocytic leukemia, GIST*		
	Nilotinib	*Chronic myelocytic leukemia, GIST*		
	Bosutinib	*Chronic myelocytic leukemia*		
	Ponatinib	*Acute lymphocytic leukemia, chronic myelocytic* *leukemia*		
Multi-targeted (ALK) tyrosine kinase inhibitors	Brigatinib	*Non-small cell lung cancer (EML4-ALK)*	Inhibits multiple receptor tyrosine kinases (ALK, HGFR, c-MET, ROS1, IGF-1R, FLT-3, EGFR, etc.), blocking cell proliferation.	Sinus bradycardia, hypertension, QTc prolongation, edema, pulmonary embolism, syncope
	Crizotinib			
	Ceritinib			
Multi-targeted (MEK) tyrosine kinase inhibitors	Cobimetinib	*Melanoma and non-small cell lung cancer (BRAF* *V600E and V600K mutations)*	MEK1 and MEK2 inhibitors (BRAF pathway), causing decreased proliferation, cell cycle arrest and apoptosis. Some also inhibit RAS, RAF, and ERK.	Cardiomyopathy, hypertension
	Trametinib			
	Vemurafenib			Peripheral edema, hypotension, atrial fibrillation, QTc prolongation, retinal vein occlusion, vasculitis
Multi-targeted (ERBB) tyrosine kinase inhibitors	Lapatinib	*Breast cancer (ERBB2+)*	Inhibits EGFR (ERBB1) and HER2 (ERBB2), regulating cellular proliferation and survival	Peripheral edema, cardiomyopathy, heart failure, hypertension, arrhythmias
	Osimertinib	*Non-small cell lung cancer (ERBB1 T790M mutation)*	Inhibits EGFR (ERBB1 T790M and L858R mutations), regulating cellular proliferation and survival	Cardiomyopathy, QTc prolongation, venous thromboembolism, stroke
Proteasome inhibitors	Carfilzomib	*Multiple myeloma*	Inhibits the 20S proteasome, leading to cell cycle arrest and apoptosis	Hypotension, acute pulmonary edema, cardiomyopathy, heart failure, cardiogenic shock, bradyarrhythmias and tachyarrhythmias, angina pectoris, cerebrovascular accident, venous thromboembolism, hemorrhagic stroke, myocardial infarction, pericardial effusion, pericarditis, peripheral edema, pulmonary embolism.
	Bortezomib	*AL amyloidosis, follicular lymphoma, mantle cell* *lymphoma, Waldenstrom macroglobulinemia, multiple* *myeloma*	Inhibits the 26S proteasome, leading to cell-cycle arrest, and apoptosis	
Antimicrotubule agents	Vinblastine	*Hodgkin lymphoma, testicular cancer, bladder* *cancer, melanoma, non-small cell lung cancer, soft* *tissue sarcoma*	Binds to tubilin and inhibits microtubulin formation; it is specific of M and S phases.	Angina, hypotension, myocardial ischemia and infarction, Raynaud’s phenomenon, limb ischemia
	Paclitaxel	*Breast cancer, bladder cancer, cervical cancer,* *endometrial cancer, esophageal cancer, gastric* *cancer, head and neck cancer, non-small cell lung* *cancer, small cell lung cancer, testicular cancer, soft* *tissue sarcoma, thymoma/thymic carcinoma, penile* *cancer, ovarian cancer*	Inhibits microtubule disassembly, interfering with the late G2 mitotic phase, and inhibits cell replication. In addition, it can distort mitotic spindles, resulting in the breakage of chromosomes.	Edema, hypotension, arrhythmias, hypertension, syncope, cardiomyopathy, heart failure, venous thrombosis
	Docetaxel	*Breast cancer, bladder cancer, bone sarcoma,* *esophageal cancer, gastric cancer, head and neck* *cancer, non-small cell lung* *cancer, small cell lung cancer, ovarian cancer, pancreatic cancer, prostate* *cancer, soft tissue sarcoma, uterine sarcoma*	Inhibits microtubule disassembly, interfering with the M mitotic phase, and inhibits cell replication	Hypotension, cardiomyopathy, heart failure. **BOX WARNING: FLUID** **RETENTION (including pulmonary** **edema)**
	Eribulin	*Breast cancer, liposarcoma*	Synthetic analogue of halichondrin B that inhibits polymerization of tubulin.	Peripheral edema, hypotension, QTc prolongation
	Ixabepilone	*Breast cancer*	Epothilone B analog, inhibits tubulin (G2/M phase inhibitor)	Peripheral edema, angina pectoris
Immunomodulators	IL-2	*Melanoma, neuroblastoma, renal cell cancer*	Promotes proliferation, differentiation, and recruitment of T and B cells, NK cells, thymocytes, LAK cells, and TILs, causing subsequent interactions between the immune system and malignant cells.	Capillary leak syndrome, acute myocarditis, hypotension, peripheral edema, cardiomyopathy, heart failure, ventricular tachyarrhythmias, cardiac arrest, myocardial infarction. **BOX** **WARNING: CARDIOPULMONARY** **DISEASE, CAPILLARY LEAK** **SYNDROME (including** **supraventricular and ventricular** **arrhythmias and myocardial infarction)**
	Interferon	*Melanoma, renal cell cancer*	Inhibits cellular growth, alters cellular differentiation and cell surface antigen expression, interferes with oncogene expression, increases phagocytic activity of macrophages, and augments cytotoxicity of lymphocytes	Chest pain, myocardial ischemia and infarction, atrial and ventricular tachyarrhythmias, edema, hypertension, cardiomyopathy, heart failure. **BOX** **WARNING: ISCHEMIC DISORDERS** **(including stroke and myocardial** **infarction)**
	Thalidomide	*AL amyloidosis, Waldenstrom macroglobulinemia,* *multiple myeloma*	Increases NK cell number and levels of IL-2 and interferon gamma. Also inhibits angiogenesis, increases cell-mediated cytotoxic effects, and alters the expression of cellular adhesion molecules.	Edema, deep vein thrombosis, hypotension, hypertension, chest pain, atrial tachyarrhythmias, myocardial infarction, pulmonary embolism, syncope, stroke, angina pectoris, cardiomyopathy, heart failure, cardiac arrest, cardiogenic shock, increased cardiac enzymes. **BOX** **WARNING: ARTERIAL AND VENOUS** **THROMBOEMBOLISM**
	Lenalidomide	*Mantle cell lymphoma, multiple myeloma, chronic* *lymphocytic leukemia, myelodisplastic syndrome,* *AL amyloidosis, renal cell cancer, non-Hodgkin* *lymphoma*	Inhibits secretion of proinflammatory cytokines; enhances cell-mediated immunity by stimulating proliferation of anti-CD3 stimulated T cells (resulting in increased IL-2 and interferon gamma secretion); inhibits trophic signals to angiogenic factors in cells.	
mTOR inhibitors	Sirolimus	*GVHD, renal angiomyolipoma*	Reduces protein synthesis and cell proliferation by binding to FKBP-12 and subsequently inhibiting mTOR activation, halting the cell cycle at the G1 phase. Also reduces angiogenesis by inhibiting VEGF and HIF-1 expression. Temsirolimus is the prodrug of sirolimus, the active metabolite. Everolimus is a sirolimus derivative.	Peripheral edema, hypertension, angina pectoris, atrial fibrillation, cardiomyopathy, heart failure, deep vein thrombosis, hypotension, pulmonary embolism, renal artery thrombosis, syncope
	Everolimus	*Breast cancer, renal cell cancer, astrocytoma, PNET*		
	Temsirolimus	*Renal cell cancer*		
Differentiation agents	Tretinoin (ATRA)	*Acute promyelocytic leukemia*	Binds to nuclear receptors, decreasing proliferation and inducing differentiation of primitive promyelocytes	Peripheral and facial edema, arrythmias, pericardial effusion/tamponade, myocardial ischemia and infarction, hypertension, cardiomyopathy, stroke, myocarditis, pericarditis, retinoic acid syndrome
	Arsenic trioxide		Induces apoptosis of primitive promyelocytes via DNA fragmentation	Tachycardia, QTc prolongation, angina, hypotension. **BOX WARNING: QTc** **PROLONGATION, TdP, AND SCD**
HDAC inhibitors	Vorinostat	*Cutaneous T-cell lymphoma*	Inhibits HDAC1, HDAC2, HDAC3, and HDAC6, resulting in the accumulation of acetyl groups, which alters chromatin structure and transcription factor activation, leading to cell growth arrest and apoptosis	Peripheral edema, QTc prolongation, hypotension, tachyarrhythmias, pulmonary embolism, hypertension. **BOX WARNING: SEVERE FATAL** **CARDIAC ISCHEMIC EVENTS AND** **ARRHYTHMIAS (panobinostat)**
	Romidepsin	*Cutaneous and peripheral T-cell lymphoma*		
	Panobinostat	*Multiple myeloma*		

### Cardiotoxic radiotherapy

The significant delay between exposure to mediastinal radiotherapy and manifestation of heart disease, reporting bias, and the frequent concomitant use of cardiotoxic chemotherapy precludes an accurate determination of the incidence of radiation-induced cardiotoxicity ^8^. Having said that, it is believed that cancer survivors who have undergone chest radiotherapy have a 23% increase in absolute risk of cardiovascular morbidity and mortality after 20 years ^22^. When considering the risk of radiotherapy-induced cardiomyopathy, for example, Hodgkin lymphoma survivors who received mediastinal radiotherapy have a fivefold increase after 30 years ^23^, whereas the greatest risk for breast cancer survivors belongs to those who received left-sided chest radiation and concomitant anthracycline chemotherapy ^24^. This laterality risk factor is likely related to the higher incidence of severe CAD in the mid and distal left anterior descending and distal diagonal arteries that is also present in this population, which could contribute to left ventricular (LV) dysfunction ^25^.

Myocardial injury induced by radiotherapy has the hallmark of increased interstitial myocardial fibrosis ^6^, which in turn leads to diastolic LV dysfunction ^26^ and subtle contractile impairment ^27^. These pathological changes may also account for the higher incidence of conduction abnormalities, cardiovascular autonomic dysfunction, impaired exercise performance, and overall mortality ^28^. Additionally, cardiac radiation is associated with complex stenotic and regurgitant valvular lesions ^29^, pericardial disease ^6^, and carotid artery disease ^30^, among other conditions.

## Stages of cancer therapeutics-induced cardiotoxicity

**Figure 1.**
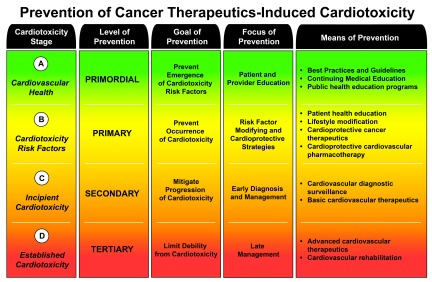
Prevention of cancer therapeutics-induced cardiotoxicity. Prevention of cancer therapeutics-induced cardiotoxicity.

Patterned after an established classification of disease progression ^31^, we have divided CTIC into four distinct stages, i.e. A, B, C, and D (see Figure 1). Stage A CTIC refers to cancer patients with cardiovascular health. Stage B CTIC designates cancer patients with high risk of developing CTIC. Risk factors for CTIC can be broadly divided into those pertaining to the patient and those pertaining to the cancer therapies implemented (see Table 2) ^5, 30, 32, 33^. Stage C CTIC denotes “incipient” cardiotoxicity; this is the early stages of the cardiotoxic process before it becomes clinically apparent. This stage is characterized by the appearance of abnormal biomarkers that precede the clearly defined diseased entities (e.g. QTc prolongation precedes Torsade de Pointes and sudden cardiac death, and sarcomeric protein or natriuretic peptide serum elevations precede LV dysfunction and overt heart failure, etc.). Finally, stage D CTIC refers to established cardiotoxicity, which is manifested by cardiovascular syndromes in early or late stages, that requires standard diagnostic modalities and medical and surgical therapies derived from expert consensus guidelines ^8, 31, 34– 36^.

**Table 2.** Risk factors of cancer therapeutics-induced cardiotoxicity. CAD, coronary artery disease; CVD, cardiovascular disease; HF, heart failure; LVEF, left ventricular ejection fraction; RT, radiotherapy; SCD, sudden cardiac death; US FDA, United States Food and Drug Administration.

Risk factors of cancer therapeutics-induced cardiotoxicity			
Patient	Age		
	Sex		
	Risk Factors of CVD	Health behaviors	Smoking/tobacco use
			Overweigth and obesity
			Physical inactivity
			Poor nutrition
		Health factors	Hypertension
			Diabetes mellitus
			Hyperlipidemia
			Metabolic syndrome
			Kidney disease
		Risk factors of SCD	QTc prolongation
			Electrolyte abnormalities
			Proarrhythmic drugs
	Pre-existent CVD		e.g. CAD, HF, arrhythmias, etc
Cancer therapies	Cardiotoxic chemotherapy	High-dose anthracycline therapy	e.g. doxorubicin ≥250 mg/m ^2^ or epirubicin ≥600 mg/m ^2^
		Low-dose anthracycline or trastuzumab therapy in high-risk patients	e.g. low normal LVEF (<53%), two or more general CVD risk factors, age 60 or over, established moderate to severe CVD
		Low-dose anthracycline and trastuzumab sequential therapy	e.g. doxorubicin <250 mg/m ^2^ or epirubicin <600 mg/m ^2^ + trastuzumab
		Other chemotherapy	e.g. US FDA box warning agents
	Cardiotoxic radiotherapy	High-dose cardiac radiation therapy	e.g. cardiac RT ≥30 Gy or ≥2 Gy/day
		Inability of cardiac avoidance	e.g. anterior or left chest radiation, tumor in cardiac proximity, lack of shielding, etc.
	Combination of cardiotoxic cancer therapies	Low-dose anthracycline + low- dose radiation therapy	e.g. doxorubicin <250 mg/m ^2^ or epirubicin <600 mg/m ^2^ + cardiac RT <30 Gy

## Levels of prevention

Preventive strategies for CTIC can also be divided into four standard levels, i.e. primordial, primary, secondary, and tertiary, which correspond with the stages of CTIC; each level of prevention has a particular goal, focus, and means (see Figure 1).

Primordial prevention is principally focused on the education of both patients and providers and on the implementation of general best practices to impede the emergence and development of risk factors for CTIC. This is being accomplished by the explosion of expert consensus guidelines in the last decade (see “expert consensus guidelines” below) as well as a growing presence of cardio-oncology programs in major oncology and cardiology scientific meetings. Moreover, there has been an increasing number of continuing medical education materials and public health education programs in this topic, all serving to raise awareness and educate on the cardiovascular effects of cancer therapies. Furthermore, the International Cardio-Oncology Society and the Canadian Cardiac Oncology Network have recently partnered in the writing of a cardio-oncology multidisciplinary training proposal to formally educate physicians in this developing field ^37^.

Primary prevention has the goal of impeding the emergence of CTIC. The diagnosis and control of modifiable risk factors (see Table 2) and the promotion of cardiovascular health in the cancer population are of utmost importance. In addition, the administration of cardioprotective therapies to selected patients with unavoidable moderate and high risk of CTIC is a means of primary prevention (see “cardioprotectants” below).

Secondary prevention is enforced once cardiac toxicity is incipient; early diagnosis and surveillance (see “blood biomarkers and diagnostic modalities” below), implementation of cardioprotective strategies, and administration of cardioprotective and basic therapies have the overarching goal to mitigate the progression of cardiotoxicity, restore cardiovascular health, and prevent complications. As in most health conditions, earlier diagnosis and treatment of CTIC seem to translate into improved outcomes ^38^. Inspired by the American Society of Clinical Oncology (ASCO) clinical practice guideline on the prevention and monitoring of cardiac dysfunction in survivors of adult cancers ^5^, as well as by other recent expert consensus guidelines that include recommendations on the prevention of CTIC ^8, 32, 39, 40^, we have constructed a table summarizing the general evidence-based recommendations for the prevention of cardiotoxicity before, during, and after cancer therapies (see Table 3).

**Table 3.** Preventive strategies for cancer therapeutics-induced cardiotoxicity. DM, diabetes mellitus; HL, hyperlipidemia; HTN, hypertension.

Preventive strategies for cancer therapeutics-induced cardiotoxicity			
Before cardiotoxic cancer therapy	Prioritize non-cardiotoxic cancer therapies without compromising cancer-specific outcomes	
	Diagnosis and control of modifiable cardiovascular risk factors (e.g. HTN, DM, HL, etc.)	
	Establish cardiovascular health (e.g. clinical examination, imaging, biomarkers)	
	Referral to specialist as appropriate	
During cardiotoxic cancer therapy	Diagnosis and control of modifiable cardiovascular risk factors (e.g. HTN, DM, HL, etc.)	
	Evaluate and maintain cardiovascular health (e.g. clinical examination, imaging, biomarkers)	
	Referral to specialist as appropriate	
	Cardiotoxic chemotherapy	Prioritize liposomal formulation and continuous infusion of doxorubicin
		Prioritize the use of dexrazoxane administration when considered appropriate (e.g. high-dose anthracyclines)
		Discontinue chemotherapy when considered appropriate
	Mediastinal radiotherapy	Prioritize lowest clinically effective radiation dose
		Deep-inspiration breath holding radiotherapy techniques
		Intensity-modulated radiotherapy
		Discontinue radiotherapy when considered appropriate
After cardiotoxic cancer therapy	Diagnosis and control of modifiable cardiovascular risk factors (e.g. HTN, DM, HL, etc.)	
	Monitor cardiovascular health (e.g. clinical examination, imaging, biomarkers)	
	Referral to specialist as appropriate	

Lastly, once CTIC has progressed sufficiently to be manifest in cardiovascular syndromes (e.g. HF, arrhythmias, acute coronary syndromes, etc.), tertiary prevention aims to limit further progression and disability, and promote rehabilitation, by both basic and advanced cardiovascular therapeutics. The evaluation and management of these defined CTIC syndromes are similar to those encountered in non-cancer patients. There are several clinical practice guidelines for the evaluation and management of these conditions in the literature ^31, 35, 36, 41, 42^, and some specifically address the cancer population ^8, 9^; these tertiary prevention strategies will not be further detailed in this work.

## Expert panel consensus guidelines

As mentioned above, the prevention of cardiotoxicity induced by cancer therapies has increasingly been the focus of several clinical cardiovascular and oncological societies, demonstrating the increasing relevance that this field has taken in the latest decade. In 2012, the European Society for Medical Oncology published a basic set of clinical practice guidelines for the prevention, monitoring, and management of CTIC ^40^. The American Society of Echocardiography and the European Society of Cardiovascular Imaging joined forces to create expert consensus guidelines for the multimodality imaging evaluation of cardiovascular complications of radiotherapy in adult patients in 2013 ^30^ as well as evaluation during and after cancer therapies in 2014 ^43^. These efforts aim to standardize the indications, acquisition protocols, definitions, limitations, and vendor variability for the different cardiac imaging modalities usually employed in the diagnosis and surveillance of CTIC. In 2016, the American Heart Association (AHA) released a comprehensive scientific statement describing the mechanism, magnitude, onset, and likelihood of direct myocardial toxicity of several anti-cancer medications, among other clinically approved drugs, “to assist healthcare providers in improving the quality of care for these patients” ^7^. In the same year, the Canadian Cardiovascular Society published a set of best practice guidelines for the management of cancer patients, focusing on the identification of the high-risk population and the detection and prevention of cardiotoxicity ^39^. This was followed by a position paper from the European Society of Cardiology summarizing the available evidence on the pathophysiology, prevention, diagnosis, therapeutic management, and long-term surveillance of the most common forms of cardiotoxicities induced by cancer therapies ^8^. Most recently, as mentioned above, the ASCO published a clinical practice guideline outlining general recommendations for the prevention of cardiac dysfunction in survivors of adult cancers ^5^. It was developed by an expert multidisciplinary physician panel using a systematic review (1996–2016) of 104 articles (meta-analyses, randomized clinical trials, and observational trials) and their clinical experience. Finally, the AHA has just published a scientific statement specifically and comprehensively dealing with the prevention of CVD in breast cancer patients, including that caused by cancer therapies ^32^.

## Cardioprotectants

The development and investigation of cardioprotective agents has been exponentially increasing since the early days of anthracycline cardiotoxicity. To date, only one cardioprotectant is approved for clinical use, i.e. dexrazoxane; many others have been tested in the clinical setting, and an even larger number are on preclinical stages of investigation (see Table 4 for a succinct list of cardioprotective agents for CTIC that have been shown to be useful at different stages of research). The vast majority of cardioprotectants have been tested in the setting of anthracycline administration, either alone or in combination with other chemotherapeutic agents; a small number has been tested in trastuzumab-only administration.

**Table 4.** Cardioprotectants in cancer therapeutics-induced cardiotoxicity. ACEI, angiotensin-converting enzyme inhibitor; ARB, angiotensin receptor blocker; MRA, mineralocorticoid receptor antagonist; NSAID, non-steroidal anti-inflammatory drug; PC-SOD, lecithinized human recombinant super oxide dismutase.

Cardioprotectants in cancer therapeutics-induced cardiotoxicity			
Clinical	Antidotes	Dexrazoxane	Lipshultz *et al*. ^49^
		N-acetylcysteine	Myers *et al*. ^54^
	Beta-blockers	Carvedilol	Avila *et al*. ^55^
		Nebivolol	Kaya *et al*. ^56^
		Bisoprolol	Pituskin *et al*. ^57^
		Metoprolol	Georgakopoulos *et al*. ^58^
	ACEIs	Enalapril	Cardinale *et al*. ^59^
		Ramipril	Jensen *et al*. ^60^
		Perindopril	Pituskin *et al*. ^57^
	ARBs	Valsartan	Nakamae *et al*. ^61^
		Candesartan	Gulati *et al*. ^62^
	MRAs	Spironolactone	Akpek *et al*. ^63^
	Statins	Atorvastatin	Acar *et al*. ^64^
	Natural supplements	Melatonin	Lissoni *et al*. ^65^
		Ubiquinone	Iarussi *et al*. ^66^
		Vitamins C and E	Wagdi *et al*. ^67^
		Levocarnitine	Waldner *et al*. ^68^
Preclinical	ACEIs	Temocapril	Tokudome *et al*. ^69^
		Delapril	Maeda *et al*. ^70^
		Zofenopril	Sacco *et al*. ^71^
	ARBs	Losartan	Matouk *et al*. ^72^
	Statins	Fluvastatin	Riad *et al*. ^73^
	Biguanides	Metformin	Kobashigawa *et al*. ^74^
	Prostacyclins	Iloprost	Neilan *et al*. ^75^
	NSAIDs	Meloxicam	Hassan *et al*. ^76^
	Vasodilators	Diazoxide	Hole *et al*. ^77^
		Molsidomine	Disli *et al*. ^78^
		Nicorandil	Ahmed *et al*. ^79^
	Iron salts	Ferric carboxymaltose	Toblli *et al*. ^80^
	Neuropeptides	Ghrelin	Wang *et al*. ^81^
	Natural antioxidants	Dihydromyricetin	Zhu *et al*. ^82^
		Hydroxytyrosol	Granados-Principal *et al*. ^83^
		Sesame oil	Saleem *et al*. ^84^
		Sesamin	Su *et al*. ^85^
		Salidroside	Wang *et al*. ^86^
		Glutathione	Mohamed *et al*. ^87^
		Quercetin	Matouk *et al*. ^72^
		Isorhamnetin	Sun *et al*. ^88^
		Cannabidiol	Fouad *et al*. ^89^
		Resveratrol	Dolinsky *et al*. ^90^
		indole-3-carbinol	Hajra e *t al*. ^91^
		α-Linolenic acid	Yu *et al*. ^92^
	Synthetic antioxidants	Didox	Al-Abd *et al*. ^93^
	Other	Mdivi-1	Gharanei *et al*. ^94^

### Dexrazoxane

In the US, dexrazoxane is the only approved cardioprotective agent consistently shown to reduce the incidence or severity of AIC ^44^. It is recommended to be given intravenously, in a 10:1 ratio of dexrazoxane:doxorubicin (e.g. dexrazoxane 500 mg/m ^2^:doxorubicin 50 mg/m ^2^) in the context of normal renal function; cardiac monitoring should be continued during dexrazoxane therapy ^17^. Its use has been associated with statistically significant risk reductions for most doxorubicin-related cardiotoxic outcomes (other than survival) ^45^, without compromising its therapeutic efficacy, in both pediatric and adult populations ^46– 49^. Although currently dexrazoxane use is strictly restricted to women with metastatic breast cancer who have received a cumulative doxorubicin dose of 300 mg/m ^2^ and need continued treatment to maintain tumor control ^44, 50^, its use in the treatment of other malignancies has been endorsed by expert guidelines ^51^. Having said that, dexrazoxane is not currently recommended for routine use with the initiation of doxorubicin therapy for either primary or metastatic disease ^51– 53^. It needs to be noted that dexrazoxane was associated with a potential increased risk of acute myeloid leukemia, myelodysplastic syndrome, and second malignant neoplasms in a pediatric population with Hodgkin lymphoma in a single study a decade ago ^95^. Many later studies have not been able to reproduce these initial results ^45, 96– 98^. Furthermore, a recent large clinical trial in a pediatric population corroborated these latter findings, suggesting that dexrazoxane was indeed cardioprotective, did not interfere with antitumor efficacy, did not result in an increased occurrence of toxicities, and had no association with a significant rise in second malignancies ^99^.

### Cardiovascular pharmacotherapy

Given their consistent benefit in other cardiovascular conditions (e.g. HF and CAD), beta-blockers, angiotensin converting-enzyme inhibitors (ACEIs), angiotensin receptor blockers (ARBs), mineralocorticoid receptor antagonists (MRAs), and HMG-CoA reductase inhibitors (statins) have been extensively studied in the clinical setting, in the context of both anthracycline and trastuzumab therapy, for the prevention of LV dysfunction.

Beta-blocker agents with antioxidant properties such as carvedilol ^100– 102^ and nebivolol ^56^ have shown the most promising results in early small clinical trials investigating their cardioprotective effects. Regrettably, in the so far largest clinical trial of beta-blockers for the prevention of cardiotoxicity under contemporary anthracycline dosage, carvedilol monotherapy had no impact on the incidence of early onset of LV ejection fraction (LVEF) reduction when compared to placebo in a breast cancer population ^55^. Similarly, ACEI monotherapy with enalapril ^59^ and ramipril ^60^ has also been shown to be beneficial in early small clinical trials; however, the administration of enalapril monotherapy either before chemotherapy or during or after chemotherapy in selected patients with elevated serum troponin levels failed to have a significant impact on outcomes in the most recent multicenter clinical trial ^103^. As for ARBs, valsartan was shown to be beneficial in small clinical trials over a decade ago ^61^; however, the use of candesartan as a cardioprotectant has recently provided conflicting results in well-conducted randomized placebo-controlled clinical trials ^104, 62^. The cardioprotective effects of spironolactone monotherapy have also been promising in early small clinical settings ^63^, but data from larger randomized clinical trials are still lacking.

Several clinical trials have investigated the cardioprotective effects of combined neurohormonal inhibition, i.e. beta-blockers plus ACEIs/ARBs, as is recommended in the general population with HF ^34^. Over a decade ago, early initiation of combined beta-blockers and ACEIs was shown to provide benefit in a small population of established AIC, albeit the effect was thought to be mediated mainly by beta-blockers ^105^. Since then, the role of combined neurohormonal inhibition in cardioprotection has been repeatedly evaluated up to this day in the settings of anthracycline, trastuzumab, or sequential chemotherapy. In the only positive trial to date, the combination of enalapril and carvedilol was shown to prevent deterioration of LV function in adult patients with hematological malignancies undergoing anthracycline therapy ^106^. However, there are significant concerns regarding this trial, including lack of blinding and differing results based on the methods used to quantify LVEF, making it difficult to conclusively interpret ^107^. In other clinical settings, metoprolol has been tested in combination with enalapril ^58^ and with candesartan ^62^, with disappointing results. Similarly, the combination of bisoprolol and perindopril failed to prevent trastuzumab-induced LV remodeling in a modern cohort of ERBB-positive breast cancer patients ^57^. Finally, in the as-yet-unpublished work by Guglin *et al*. presented at the 2018 American College of Cardiology annual meeting, both lisinopril and carvedilol failed to prevent cardiotoxicity in breast cancer patients treated with trastuzumab monotherapy, whereas both drugs prevented cardiotoxicity in patients who received both anthracycline and trastuzumab sequential therapy ^108^.

The cardioprotective role of statins has also been evaluated in small retrospective and prospective analyses, both with non-specific statins ^109, 110^ and atorvastatin monotherapy ^64^, and was found to be beneficial. These findings are very promising but are yet to be corroborated in larger randomized placebo-controlled trials (simvastatin NCT02096588; atorvastatin NCT02674204).

### Natural supplements

Clinical cardioprotective data involving natural supplements are scarce but growing. Ubiquinone (coenzyme Q10) administration in children receiving anthracyclines was associated with a lesser degree of LV dysfunction and remodeling ^66^. N-acetylcysteine, administered either alone or with vitamins E and C, averted LV dysfunction from developing in patients receiving high-dose doxorubicin and/or radiotherapy, respectively ^67, 54^. Melatonin ^65^ and levocarnitine ^68^ have also been tested in the clinical setting with positive results. Larger randomized placebo-controlled trials are lacking as to draw firm conclusions relevant to the clinical practice.

### Preclinical agents

Many other agents have been shown to ameliorate anthracycline cardiotoxicity in small animal models of CTIC. Clinically available agents such as losartan ^72^, fluvastatin ^73^, metformin ^74^, iloprost ^75^, and meloxicam ^76^ as well as other clinically unavailable ACEIs ^69– 71^ have been shown to have cardioprotective results *in vivo*. Vasodilators ^77– 79^, neuropeptides ^81^, and iron salts ^80^ have also been found to be useful. Finally, given that the pathogenesis of anthracyclines is in part related to increased oxidative stress ^100^, several natural antioxidants (e.g. sesamin ^85^ and sesame oil ^84^ and hydroxytyrosol ^83^, among others ^82, 86– 92^) have been tested and shown various degrees of cardioprotective effects. Didox, a synthetic antioxidant, was also shown to significantly potentiate the cytotoxicity of doxorubicin in liver cancer cells while at the same time protecting the murine model from cardiotoxicity ^93^. Mdivi-1, a mitochondrial division/mitophagy inhibitor, was also shown to lessen AIC ^94^.

### Other cardioprotective strategies

Within a family of cardiotoxic agents, there are variations in terms of cardiac safety. For example, the use of pegylated liposomal doxorubicin has been associated with a lower incidence of CTIC and HF ^111, 112^. Similarly, epirubicin or mitoxantrone are also believed to cause less cardiotoxicity compared with doxorubicin ^113^. When considering the large family of multitargeted tyrosine kinase inhibitors, vandetanib, nilotinib, and ponatinib seem to possess the highest cardiotoxicity risk ^17^. The role of exercise therapy in the prevention of CTIC remains controversial because of conflicting results ^114, 115^.

In summary, with the exception of dexrazoxane, no conclusive recommendations can be made on the clinical use of cardioprotectants for either stage B or stage C CTIC ^5^.

## Blood biomarkers

Blood biomarkers, in particular myocardial natriuretic peptides (i.e. NTproBNP and BNP) and sarcomeric proteins (i.e. troponin I and T), have been an integral part of the diagnostic and prognostic armamentarium in common cardiovascular conditions, such as HF and CAD. As it would seem natural, they have been progressively adopted in clinical practice to assist in the diagnosis or surveillance of patients with incipient and established CTIC, in particular LV dysfunction and HF (see Table 5 for a list of various clinical and preclinical biomarkers shown to predict CTIC) ^5^.

**Table 5.** Blood biomarkers in cancer therapeutics-induced cardiotoxicity. ANP, atrial natriuretic peptide; BNP, B-type natriuretic peptide; cMLC1, cardiac myosin light chain-1; cTnAAbs, cardiac troponin specific autoantibodies; cTnI, cardiac troponin I; cTnT, cardiac troponin T; GWAS, genome-wide association study; hs-CRP, high-sensitive C-reactive protein; hs-TnI, high-sensitive troponin I; GDF15, growth differentiation factor-15; GPBB, glycogen phosphorylase BB; IMA, ischemia modified albumin; MPO, myeloperoxidase; NTproBNP, amino-terminal pro B-type natriuretic peptide; PlGF, placental-derived growth factor; ROS, reactive oxygen species.

Blood biomarkers in cancer therapeutics-induced cardiotoxicity			
Clinical	Myocardial natriuretic peptides	NTproBNP	De Iuliis *et al*. ^119^
		BNP	Lenihan *et al*. ^37^
		ANP	Nousiainen *et al*. ^120^
	Myocardial sarcomere proteins	cTnI	Cardinale *et al*. ^117^
		cTnT	Kilickap *et al*. ^118^
		hs-cTnI	Sawaya *et al*. ^121^
		hs-cTnT	Katsurada *et al*. ^122^
		us-cTnI	Ky *et al*. ^123^
	Other biomarkers	cTnAAbs	Ylänen *et al*. ^124^
		Hb	Garrone *et al*. ^125^
		hsCRP	Onitilo *et al*. ^126^
		MPO	Ky *et al*. ^123^
		PIGF	Putt *et al*. ^127^
		GDF15	Arslan *et al*. ^128^
		Arginine-NO metabolites	Finkelman *et al*. ^129^
		GPBB	Horacek *et al*. ^130^
		ROS	Mercuro *et al*. ^131^
		IMA	Ma *et al*. ^132^
	Single nucleotide polymorphims (GWAS)	rs2229774	Aminkeng *et al*. ^133^
		rs1786814	Wang *et al*. ^134^
		rs28714259	Schneider *et al*. ^135^
Preclinical	DNA	Doxorubcin DNA adducts	Hahm *et al*. ^136^
		Spp1, Fhl1, Timp1, Ccl7 and Reg3b	Mori *et al*. ^137^
	MicroRNA	miR-34a	Desai *et al*. ^138^
		miR-34c	Vacchi-Suzzi *et al*. ^139^
		miR-146a	Horie *et al*. ^140^
	Proteins	S100A1	Eryilmaz *et al*. ^141^
		cMLC1	ElZarrad *et al*. ^142^
		Cathepsin B	Bao *et al*. ^143^
	Proteomics pattern diagnostics		Petricoin *et al*. ^144^
	Metabolomics pattern diagnostics		Li *et al*. ^145^
	Transcriptome profiling		Todorova *et al*. ^146^

Troponin I ^59, 116, 117^ and troponin T ^118^ have been shown to be clinically useful in several clinical trials of cardiotoxicity prediction. Modern, more-sensitive assays of troponin I and T (high-sensitivity and ultra-sensitivity) have also been shown to be clinically predictive of CTIC ^121– 123^. Early studies have suggested that troponin I elevation predicted severity of CTIC ^116, 117^, and refractoriness to HF therapy in the case of trastuzumab-induced cardiomyopathy ^117^, but response to enalapril monotherapy in the case of AIC ^59^. However, in a recent large multicenter randomized clinical trial, these findings could not be corroborated ^103^. Interestingly, the presence of troponin-specific autoantibodies also predicted cardiac dysfunction by cardiac magnetic resonance (CMR) imaging in the absence of elevated traditional troponin levels ^124^. Myocardial natriuretic peptides, such as NTproBNP ^119^, BNP ^147^, and ANP ^120^, have also been shown to be clinically useful predictors of CTIC, albeit to a lesser extent.

Although the use of these blood biomarkers is currently recommended in the evaluation and surveillance of patients with CTIC ^5, 8^, their helpfulness remains disputed owing to inconsistent results in terms of sensitivity, accuracy, and reliability ^148^. Hence, various other alternative blood biomarkers have been studied in recent years, either alone or in combination, and shown also to be clinically predictive of CTIC, e.g. hsCRP ^126^, MPO ^123^, and arginine-NO metabolites (arginine, citrulline, ornithine, asymmetric dimethylarginine, symmetric dimethylarginine, and N-monomethylarginine) ^129^, among others ^125, 127, 128, 130– 132^. Likewise, many other predictive biomarker strategies are currently being developed in the preclinical arena. Proteomics ^144^ and metabolomics ^145^ pattern diagnostics, as well as transcriptome profiling ^146^, have been shown to be useful in animal models of AIC as well as the detection of doxorubicin DNA adducts (HM-dUMP, 8-OH-dGMP, HM-dCMP, and Me-dCMP) ^136^ and other particular genes that are overexpressed during incipient cardiotoxicity ^137^. Cellular proteins such as S100A1 ^141^, cMLC1 ^142^, and cathepsin B ^143^ have also been shown to have predictive value. Some microRNAs (e.g. miR-34a ^138^, miR-34c ^139^, and miR-146a ^140^) have been shown to be useful in predicting CTIC in small animal models; however, a recent clinical trial involving miR-208a measurement in breast cancer patients failed to have a predictive impact ^149^.

Finally, research efforts to identify the genetic susceptibility of AIC have been increasing in the last decade, with the purpose of risk stratifying patients before they receive anthracycline chemotherapy. To date, three main single-nucleotide polymorphisms (SNPs: rs28714259 ^135^, rs1786814 ^134^, and rs2229774 ^133^) have been identified as being strongly associated with AIC by means of genome-wide association studies (GWAS) from pediatric and adult case-controlled clinical trial populations.

## Diagnostic modalities

Non-blood diagnostic modalities are also an integral part of the evaluation of CVDs. For the purpose of early diagnosis and surveillance of CTIC, several imaging modalities have been studied since the late 1970s and shown to be of value (see Table 6). Historically, electrocardiography ^150^ was used to diagnose arrhythmias during anthracycline infusion, and radionuclide cineangiography (MUGA) ^151, 152^ was the first technique used to detect falls in LV systolic function in patients receiving anthracyclines ^153^. Although MUGA is still considered widely available and highly reproducible, it carries the main disadvantage of submitting cancer patients to small, but potentially significant, radiation exposure (5–10 mSv) ^30, 43^. Additionally, 2D-echocardiogram ^154^ and stress 2D-echocardiogram ^155^ have been shown to be beneficial in the serial evaluation of cancer patients undergoing cardiotoxic chemotherapies. Newer echocardiographic modalities, such as 3D-echocardiography ^156^ and LV global longitudinal strain (LVGLS) measurement by speckle-tracking echocardiography (STE) ^157^, have demonstrated superiority over 2D-echocardiography in terms of reproducibility and predictability, respectively. CMR is currently considered the gold standard modality in the assessment of LV and right ventricular volumes and function ^158^. Secondary modalities such as CMR strain imaging ^159^, T1 mapping ^160^, and extracellular volume fraction (ECV) ^161^ have also been clinically studied in recent years and found to be of great value in the assessment of subclinical cardiotoxicity. Among various non-imaging techniques, cardiopulmonary exercise testing was shown to detect abnormalities in peak oxygen consumption in cancer patients with apparently normal LV function ^162^, suggesting subclinical impairments of contractile reserve and chronotropic incompetence ^28^. Finally, many other imaging modalities are currently being studied in the preclinical arena to help detect incipient cardiotoxicity with high specificity and sensitivity. For example, 18F-labeled tetrapeptide caspase positron emission tomography (PET) is able to specifically diagnose doxorubicin-induced myocardial apoptosis in a murine model by detection of overexpressed myocardial caspase 3 resulting from anthracycline chemotherapy ^163^.

**Table 6.** Diagnostic modalities in cancer therapeutics-induced cardiotoxicity. 2D, two-dimensional; 3D, three-dimensional; 99m Tc, technetium-99; CMR, cardiac magnetic resonance; CPET, cardiopulmonary exercise testing; ECG, electrocardiogram; ECV, extracellular volume fraction; LVEF, left ventricular ejection fraction; LVGLS, left ventricular global longitudinal strain; MUGA, multigated acquisition; PET, positron emission tomography; RBC, red blood cells.

Diagnostic modalities in cancer therapeutics-induced cardiotoxicity		
Established clinical	ECG	Steinberg *et al*. ^150^
	MUGA (99m Tc-labeled RBC)	Schwartz *et al*. ^151^
	Stress MUGA	McKillop *et al*. ^152^
	2D-echocardiography	Thavendiranathan *et al*. ^154^
	Stress 2D-echocardiography	Khouri *et al*. ^155^
	CPET	Jones *et al*. ^162^
Novel clinical	3D-echocardiography	Walker *et al*. ^156^
	Speckle-tracking echocardiography (LVGLS)	Negishi *et al*. ^157^
	CMR	Armstrong *et al*. ^158^
	CMR strain imaging	Drafts *et al*. ^159^
	CMR T1 mapping	Lightfoot *et al*. ^160^
	CMR ECV	Jordan *et al*. ^161^
Preclinical	PET (18F-labeled tetrapeptidic caspase)	Su *et al*. ^163^

According to current guidelines, echocardiography (ideally 3D-echocardiography) is the method of choice for the evaluation of patients before, during, and after cancer therapies ^43^. CMR and MUGA scan (in that order) should be utilized as alternative modalities whenever the echocardiographic image quality is deficient ^5^. When available, measurement of LVGLS by STE is also recommended as a complementary modality ^5^. CMR should also be considered for the evaluation of chronic “constrictive” pericarditis, when the diagnosis remains uncertain after a careful echocardiographic evaluation ^43^.

To date, there is little evidence to guide the indication, timing, and frequency of use of imaging modalities in patients undergoing cancer therapies. The ASCO expert consensus recommends an echocardiographic evaluation prior to the initiation of potentially cardiotoxic cancer therapies ^5^. Routine imaging surveillance in asymptomatic patients should be offered to patients based on the healthcare provider’s perceived risk of CTIC, and the frequency of it needs to be individualized based on clinical judgment and patient circumstances ^5^. Subsequent to cardiotoxic cancer therapies, it is recommended that high-risk patients undergo a follow up LVEF evaluation between 6 and 12 months after completion of therapy ^5^.

## Conclusions

In this work, we have attempted to comprehensively and concisely survey the most relevant available literature pertaining to cardioprotection during cancer therapy. We have briefly summarized the pathophysiology of CTIC, describing the mechanisms of cardiotoxicity of various agents, and risk factors that promote this phenomenon. For didactic purposes, we have classified CTIC into four progressive stages, in which four levels of prevention are applied, each having a specific goal, focus, and means of prevention. We have subsequently reviewed the available data on cardioprotective agents, blood biomarkers, and imaging diagnostic modalities, which are the core of primary and secondary prevention strategies. Finally, we have provided general evidence-based preventive recommendations for CTIC following the most current expert consensus guidelines. The promotion of the cardiovascular health of cancer patients and cancer survivors is paramount, requiring the diligent and knowledgeable effort of a multidisciplinary team of healthcare providers; as in all medical disorders, prevention is better than cure.
